# Impact of mass distribution on insecticide-treated net coverage and usage in three high burden malaria provinces of Papua New Guinea

**DOI:** 10.1186/s40249-026-01494-x

**Published:** 2026-09-14

**Authors:** John B. Keven, Melanie Koinari, Christine Pombreaw, Micah Muri, Celine Figa, Clara Are, Solomon Lagur, John Cowan, Beatrice R. Egid, Jacob Kisomb, Petrina Johnson, Maria Ome-Kaius, Lincoln Timinao, Leanne J. Robinson, Moses Laman, Stephan Karl

**Affiliations:** 1 Vector-Borne Disease Unit, Papua New Guinea Institute of Medical Research, Madang, Papua New Guinea; 2 Australian Institute of Tropical Health and Medicine, James Cook University, Smithfield, QLD Australia; 3 Independent Global Health Research Consultant, Oxford, UK; 4 Papua New Guinea National Department of Health, National Capital District, Port Moresby, Papua New Guinea; 5 Burnet Institute, Parkville, Melbourne, Australia

**Keywords:** Bed net, Coverage, Distribution, Household, Insecticide treated net, Malaria, Control, Ownership, Usage

## Abstract

**Background:**

Papua New Guinea (PNG) completed five nation-wide mass distributions of insecticide-treated nets (ITNs) in the last 15 years. Despite this considerable achievement, malaria cases in PNG are rising. Recent national-level surveys indicate unusually low ITN coverage and usage indicators, raising concerns about the continued effectiveness of this core intervention. Significant knowledge gaps remain around factors impacting ITN effectiveness in PNG, limiting interpretation of the role of ITNs in the context of rising malaria cases. This study assessed how ITN ownership and usage indicators in three of the highest burden provinces changed after the most recent mass distribution in 2024.

**Methods:**

Cross-sectional surveys involving a target of 60 randomly selected households per village were conducted in 12 villages in East Sepik, West Sepik and Madang province and collected data on 5671 individuals belonging to 815 households. The surveys were conducted within 1–4 months before (September–December 2023), and 5–7 months after (May–October 2024) programmatic ITN mass distribution. Data were collected through household questionnaires, visual inspection of all ITNs present in households, and matching of ITNs to individual users. Household-level and person-level indicators of ITN ownership and use were evaluated using generalised linear mixed effects models.

**Results:**

Distribution targets were achieved in two provinces but were missed by 38–42% in the third province. Overall, 94.8% of houses owned at least one ITN, before, and 99.4% after distribution (*P* < 0.001). The mean number of ITNs per person rose from 0.55 to 0.64 (*P* < 0.001), and universal coverage increased from 60.0% before to 69.9% after distribution (*P* = 0.002). ITN distribution resulted in 61.1% of new ITNs in use and 55.5% of old ITNs lost to follow-up at the post distribution survey. Distribution increased ITN usage evenly across genders and ages and did not change existing overall age- or gender-related usage and sharing patterns.

**Conclusions:**

ITN ownership and use are high and sustained by 3-yearly distributions in the three provinces. ITN use was primarily driven by availability, with no further increases observed beyond universal coverage. Results also highlight the importance of post-distribution surveillance to confirm that programme targets are achieved.

**Graphical Abstract:**

**Figure:**
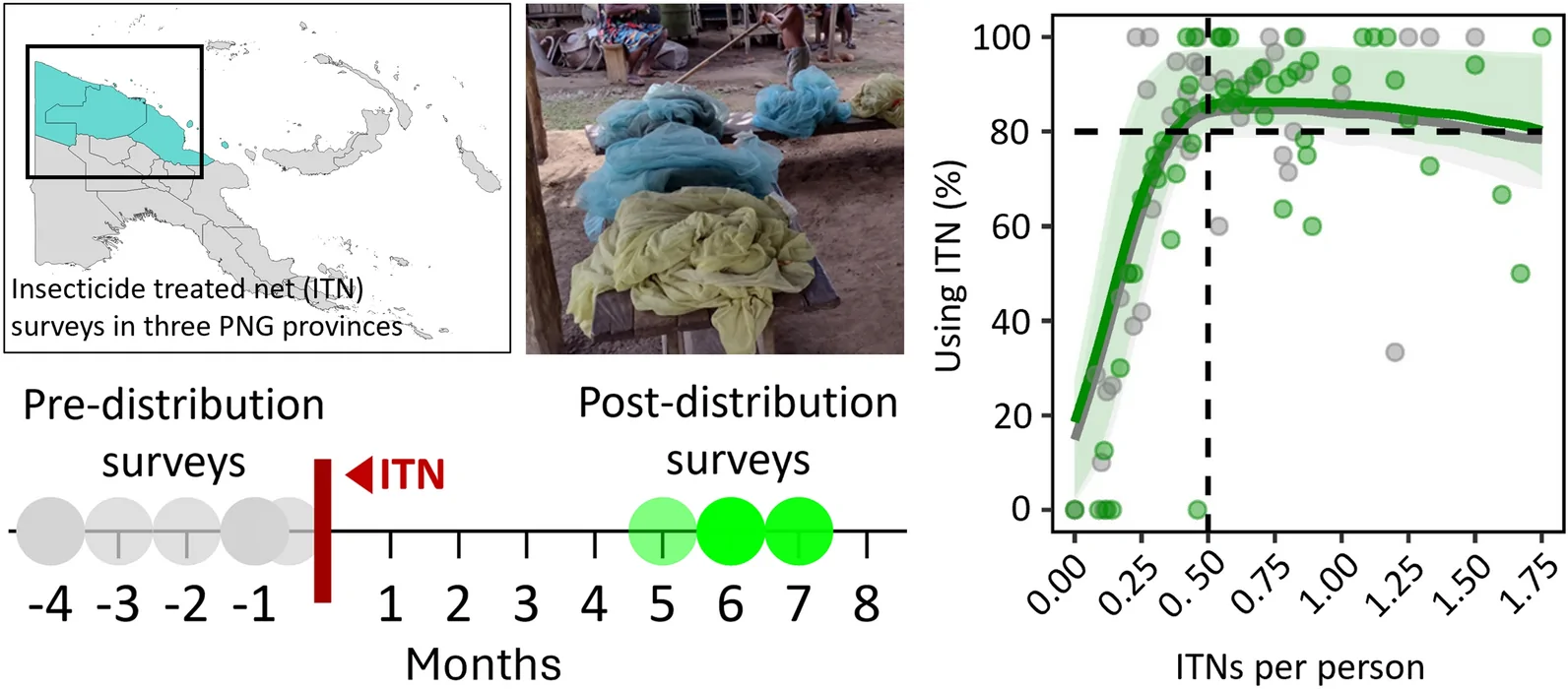

**Supplementary Information:**

The online version contains supplementary material available at https://doi.org/10.1186/s40249-026-01494-x.

## Background

Papua New Guinea (PNG) reported close to one million malaria cases in 2024. Almost half of these (416,000 cases; 43%) occurred in just three of PNG’s 22 provinces—Madang, East Sepik and West Sepik [1]. This represents more than one-third (36%) of all estimated malaria cases in the World Health Organisation (WHO) Western Pacific Region [1, 2]. Controlling malaria in these three PNG provinces alone would therefore substantially advance the region’s malaria elimination agenda [3].

Like all areas with significant malaria transmission in PNG, these high-burden provinces are part of the national mass distribution programme of insecticide-treated nets (ITNs). Coordinated by the National Department of Health’s Malaria Control Program (NMCP), more than 20 million ITNs have been delivered since 2009 [4, 5]. Although distributions occur at different times across the country, they are implemented on a triennial cycle. ITNs distributed in PNG by the NMCP are WHO prequalified products.

Despite multiple rounds of ITN distribution and amidst efforts to introduce additional and sub-nationally tailored malaria control strategies [6], it is not well understood what the triennial ITN mass distribution campaigns in PNG are achieving in terms of maintaining ITN coverage and usage, let alone in terms of their epidemiological impact [7]. With the first ITN durability studies in PNG completed only in 2024 [8], considerable knowledge gaps also persist regarding the lifespan and duration of insecticidal efficacy of ITNs in PNG. These concerns are further aggravated by evidence of unacceptably low duration of insecticidal efficacy of ITN products distributed in recent years [9–11].

Earlier analyses of determinants of ITN ownership and use were conducted during the initial stages of the national ITN mass distribution scale-up between 2008 and 2011[12, 13]. These studies identified age, gender, geographic location, and household wealth indicators as important predictors of ITN use: children were much more likely to sleep under an ITN than adults, women more likely than men, and residents of higher-quality houses were less likely to use ITNs.

No previous study in PNG has directly examined changes in ITN ownership, usage, and sharing immediately before and after a mass distribution in settings where households are already near-saturated with ITNs following repeated distribution campaigns. This study was therefore conducted to assess how key ITN ownership and usage indicators changed after the most recent mass distribution in three of PNG’s highest malaria burden provinces. It was motivated by the hypothesis that ITN use behaviours may have changed over the past 15 years of continuous nationwide mass distributions. In addition, it was prompted by increasing reports of declining ITN ownership and use in several of PNG’s highest-burden provinces despite ongoing large-scale distribution efforts.

PNG’s ITN mass distribution programme has been regularly evaluated through Malaria Indicator Surveys (MIS). Six MIS rounds have been conducted since 2008, approximately every three years, providing national-level snapshots of ITN indicators from 4–6 randomly selected villages in nearly all provinces [14]. Recent MIS results have documented declining ITN coverage and usage across PNG, with the most recent survey preceding the present study estimating that only 56% of households owned at least one ITN and that average ITN use was just 34% in the historically high ITN-use provinces of Madang, East Sepik and West Sepik [15].

MIS, however, primarily report Global Fund-defined indicators and do not examine the determinants of ITN use in detail. Additionally, MIS are not scheduled relative to local ITN distribution campaigns, meaning that province-level estimates may fall anywhere within the three-year distribution cycle and can therefore be difficult to interpret. We are not aware of a meta-analysis aligning MIS ITN indicators with the timing of ITN distributions in PNG having been published to date.

To address these gaps, the present study conducted two consecutive rounds of cross-sectional household and ITN surveys—approximately 1–4 months before, and 5–7 months after, the most recent ITN mass distribution campaigns in Madang, East Sepik and West Sepik. The aim was to better understand how programmatic ITN distributions in PNG influence ITN ownership, usage, and sharing; assess whether distribution targets are met at community, household, and individual levels; and investigate recent MIS reports suggesting low levels of ITN ownership and use.

## Methods

### Survey methods and study sites

**Fig. 1:**
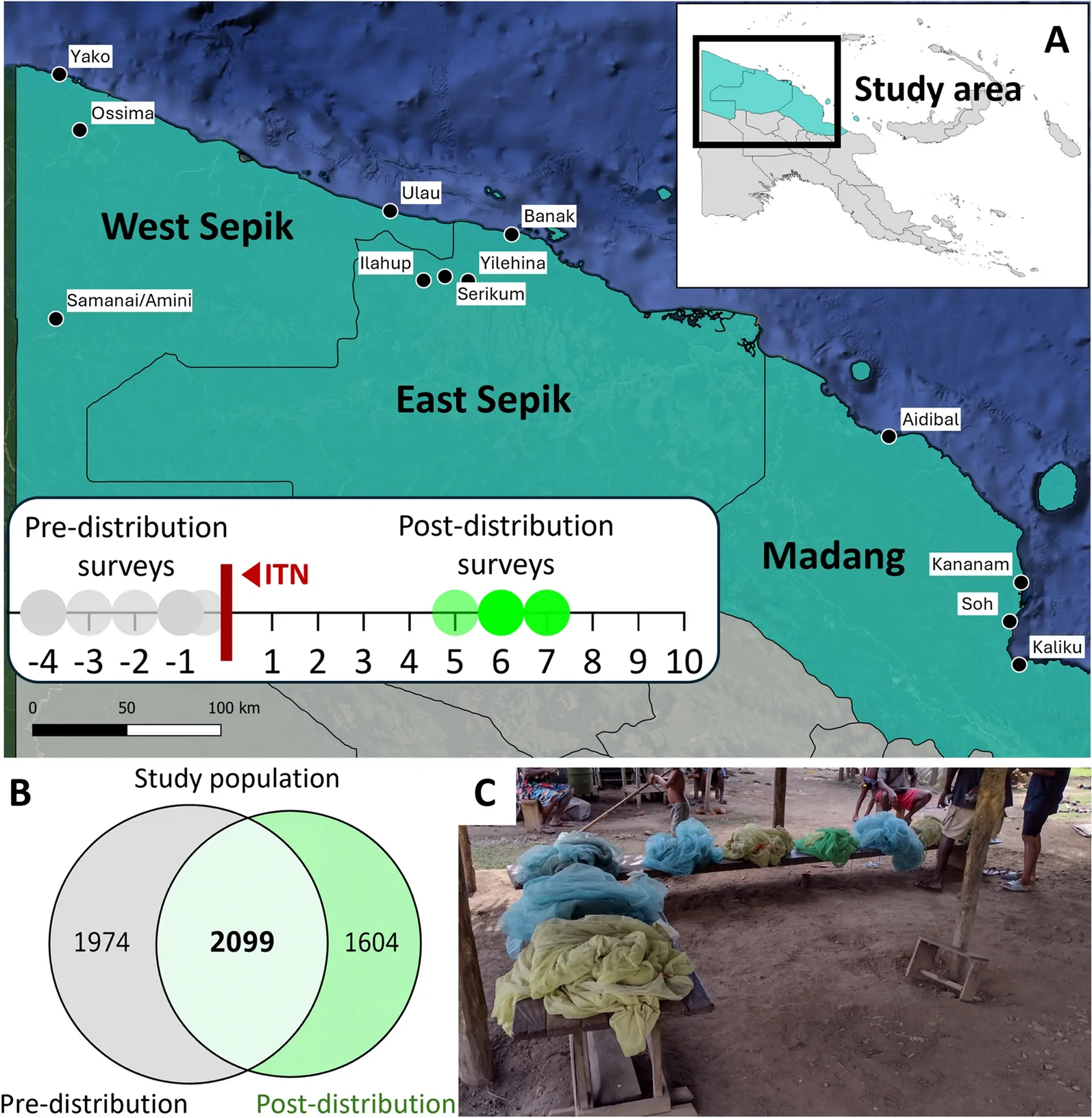
Study sites and design. **A** shows the location of the study villages, including an inset indicating the three study provinces within Papua New Guinea (PNG), and a timeline illustrating the timing of the baseline and follow-up surveys relative to the insecticide treated net (ITN) mass distribution campaigns. **B** presents the partially overlapping person-level dataset, showing the numbers of individuals surveyed at each time point. **C** provides a visual impression of the field survey activities

Cross sectional surveys were conducted in 12 villages in Madang (*n* = 4), East Sepik (*n* = 4) and West Sepik (*n* = 4) provinces of PNG (Fig. 1A) once before and once after programmatic ITN distribution. The target sample size was 60 households per village, corresponding to 240 households per province and 720 households overall per survey round for the two partially overlapping survey rounds. Under a conservative assumption of 50% prevalence for binary ITN indicators, this sample size provided 80% power at α = 0.05 to detect absolute pre-post differences of approximately 13 percentage points at province level and 7 percentage points overall.

Study village selection was based on the most recent pre-distribution census data provided by Rotarians Against Malaria (RAM) PNG, which conducts household censuses prior to each ITN distribution campaign. Villages were selected through stratified random sampling across the catchment areas of four pre-identified health facilities per province where ITNs had been distributed, in coordination with RAM and the respective Provincial Health Authorities. One village was selected from the catchment area of each health facility, resulting in a total of 12 study villages.

The study villages had an average of 131 households (range: 66–300). At baseline, an average of 54% of households per village were surveyed (range: 20–89%), compared with 50% at follow-up (range: 19–80%).

Using this approach, a total of 815 households (average 68, range 58–79 per village) were surveyed between September 2023 and October 2024. Of these, 570 were visited twice, 143 were visited only at baseline and 102 were visited only at follow-up. The first survey was conducted between 4 months and 2 weeks before the ITN mass distribution (i.e., 3 years after the previous ITN distribution) and the second survey was conducted within 5–7 months after the ITN mass distribution (Fig. 1A). The exact survey dates for each village are given in the Supporting Information (Supplementary file 1, Table S2).

### Field surveys

The survey methodology was identical at both time points. Interviews were conducted with the household head or, in their absence, another adult household member with decision-making authority. Information on all household members was provided by this respondent. Individual interviews with other household members were not conducted.

All ITNs present in a household were visually inspected for presence and properties and matched to their respective users (Fig. 1C). Information on the use of each individual ITN was collected through discussion with the present household members. ITN source was verified by inspecting ITN labels for distributor, product type and manufacturing year and cross-checking this information with available distribution data.

Data collected included whether each ITN was currently in use, who in the household was using it, whether it was shared, and, if so, among whom. For ITNs not used, the reason for non-use were recorded, including if ITNs were being reserved for later use. Demographic information for all individuals living in surveyed households were collected and linked to the ITNs that each person was using.

### Data analysis

Data analyses involved calculating basic household-level univariate statistics for ITN indicators and how they changed with ITN mass distribution, including: (i) the percentage of households owning at least one ITN, (ii) the number of ITNs per person and (iii) the proportion of households with ‘universal coverage’ (i.e., ITNs per person ≥ 0.5). Where appropriate, measures of uncertainty such as 95% confidence intervals (*CI*) of proportions, and odds ratios were calculated. These analyses did not consider repeated measures (i.e., each survey time point was treated as an independent cross-sectional survey).

The expected number of ITNs according to the NMCP distribution logic was calculated based on household composition at baseline and compared to the number of ITNs households had received at follow-up. The distribution logic is:

- 1 ITN per every father and mother + 2 children under the age of 6 years
- 1 ITN for every ≤ 3 girls and ≤ 3 boys aged 6–17 years.
- 1 ITN for every ≤ 3 additional male and female adults

Person-level data needed to be matched prior to analysis as the independent cross-sectional surveys produced partially repeated-measures data with data for some households, ITNs and individuals occurring twice, while others were observed only once, either before or after the ITN distribution. Because of manual data entry, spelling variation, and ambiguity in PNG names, repeated observations were identified using probabilistic record linkage based on the Fellegi-Sunter framework implemented in Python 3.13 (Python Software Foundation, Wilmington, Delaware, USA). Matching relied primarily on Jaro-Winkler name similarity, with additional weights for agreement in gender and age, and deterministic household blocking to restrict candidate pairs. A match-probability threshold of 0.75 was applied, which visual inspection confirmed to produce effectively complete and accurate matches. When ages differed within a matched pair, their mean was used; when genders differed (rare), the baseline gender entry was retained. Explicit ITN matching between both time points was not conducted.

The probability that an individual used an ITN the night before the survey was estimated using a Bayesian generalized linear mixed-effects model with a Bernoulli likelihood and logit link. Fixed effects included age, ITNs per person (ITNPP), survey time-point (pre- vs. post-distribution), household size, gender, and an interaction between age and time-point. Age and ITNPP exhibited clearly non-linear relationships with ITN use. Therefore, age was modelled using natural cubic splines with four degrees of freedom (DOF) and ITNPP with natural cubic splines with three DOF, to allow for flexible, non-linear relationships with ITN use without imposing restrictive parametric assumptions. Other interactions (e.g., age × gender) were evaluated but excluded due to lack of evidence for effect modification. The model accounted for within household and within village clustering of observations through a hierarchical random effects structure. Random intercepts were included for household, person, and village, and village additionally received a random slope for time-point to allow pre/post effects to vary geographically.

A closely related model was used to estimate the probability that ITN users shared an ITN the previous night. This model specified age using natural cubic splines with five DOF and ITNPP with two DOF, and included fixed effects for time-point, household size, gender, and an interaction between age and gender, to capture the marked differences in sharing patterns between males and females across age groups, while retaining model parsimony. Random-effect structures matched those of the ITN-use model. Detailed descriptions of these statistical models along with information on survey instrument and household questionnaires are presented in Supplementary file 1 (Equation S1-S2, Tables S3-S7).

The models were fit using Stan called via *brms* package in R 4.6.0 (R Core Team, Vienna, Austria). Fitting involved four chains with 3000 iterations each (1000 warm-up). Results are reported as odds ratios where appropriate or graphically for non-linear predictors, with uncertainty expressed using 95% Bayesian credible intervals (95% *CI*) and posterior tail-area probabilities (PTAP, P*).

## Results

### Summary of the surveyed population

Data on 5671 individuals were collected from interviews with the heads of 815 households across both surveys. Of these, 2099 individuals (37.0%) from 559 households (68.6%) were represented at both time points. At baseline, data were collected on 4073 individuals in 713 households, and at follow-up on 3703 individuals in 672 households (Fig. 1B). Across villages, the mean number of individuals whom data were collected was 473 (range 324–698) per village across both time points. In total 2011 ITNs were inspected at baseline and 2283 ITNs were inspected following the mass distribution.

**Fig. 2:**
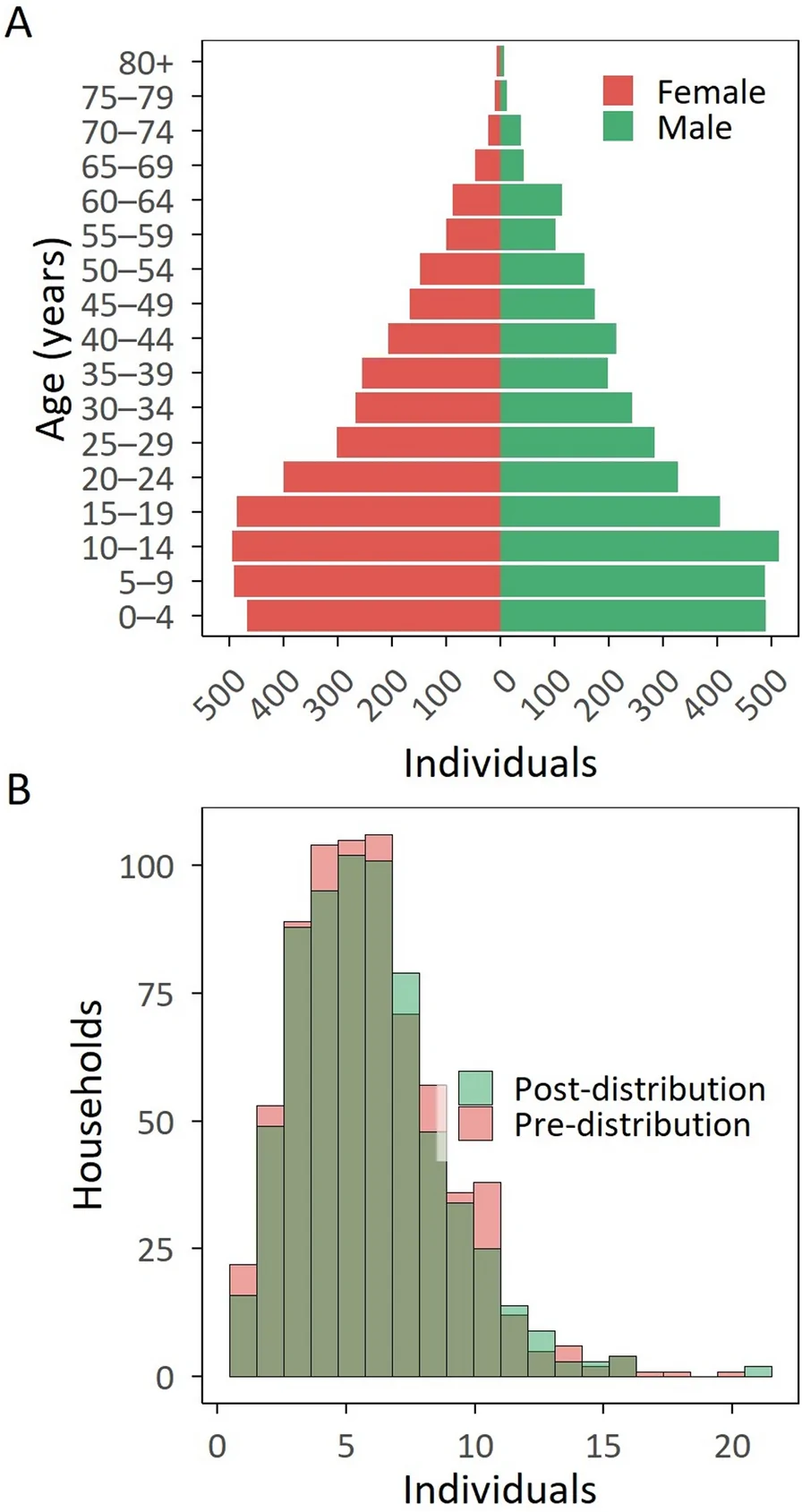
Age, gender and household size distribution of the study population. **A** shows the age and gender distribution for 5671 individuals. For individuals surveyed twice, when age records were different between baseline and follow-up, the average was calculated. When gender was different (rare), baseline gender was used. **B** shows the distribution of household size. Separate distributions for baseline (*n* = 713) and follow up (*n* = 672 households) are provided

The detailed age and gender distribution of the study population and household size (number of people in a household) are shown in Fig. 2. The median age of the study population was 20 (2.5–97.5% quantiles: 1–64) and the male to female ratio was 0.96 (Fig. 2A). Household sizes ranged from 1–21 people per household, with most houses having 4–6 people per household (Fig. 2B).

### Household-level ITN indicators

Table 1 summarises changes in ITN indicators (proportion of households with ≥ 1 ITN, mean number of ITNs per person, and proportion of households with universal coverage) before and after the mass distribution stratified by province. Corresponding raw data is presented in Supporting Information Table S1. Overall, the mass ITN distribution led to substantial improvements in ITN coverage indicators. The proportion of households owning at least one ITN increased from 94.8% to 99.4% (*P* < 0.001), the mean number of ITNs per person (averaged across households) rose from 0.55 to 0.64 (*P* < 0.001), and universal coverage improved from 60.0% to 69.9% (*P* = 0.002). Improvements were smaller in East Sepik Province—where universal coverage decreased slightly from 70.3% to 66.5%—potentially reflecting less comprehensive distribution efforts compared with the other two provinces.

**Table 1:** Household-level univariate model estimates for insecticide-treated net (ITN) indicators of household owning at least one ITN, the number of ITNs per person and the proportion of households with universal coverage

Province	Timepoint	At least 1 ITN		ITNs per person		Universal coverage	
		% (95% *CI*)	*OR* (95% *CI*) *P-*value	Mean (95% *CI*)	*P-*value	% (95% *CI*)	*OR* (95% *CI*) *P-*value
East Sepik	Baseline (*n* = 239)	97.1 (94.1–98.8)	2.13 (0.54–8.35), *P* = 0.27	0.60 (0.55–0.65)	< 0.87	70.3 (64.1–76.0)	0.84 (0.56–1.25), *P* = 0.39
	Follow-up (*n* = 215)	98.6 (96.0–99.7)		0.61 (0.56–0.65)		66.5 (59.8–72.8)	
Madang	Baseline (*n* = 234)	97.4 (94.5–99.1)	6.13 (0.73–51.33), *P* = 0.06	0.59 (0.55–0.63)	< 0.001	62.4 (55.8–68.6)	1.87 (1.26–2.79), *P* = 0.002
	Follow-up (*n* = 234)	99.6 (97.6–100.0)		0.72 (0.67–0.77)		75.6 (69.6–81.0)	
West Sepik	Baseline (*n* = 240)	90.0 (85.5–93.5)	50.60*** (3.06–837.00), *P* < 0.001	0.46 (0.42–0.50)	< 0.001	47.5 (41.0–54.0)	2.27 (1.56–3.31), *P* < 0.001
	Follow-up (*n* = 223)	100.0 (98.4–100.0)		0.60 (0.56–0.64)		67.3 (60.7–73.4)	
Overall	Baseline (*n* = 713)	94.8 (92.9–96.3)	9.14 (3.24–25.79), *P* < 0.001	0.55 (0.53–0.58)	< 0.001	60.0 (56.3–63.6)	1.55 (1.23–1.95), *P* = 0.002
	Follow-up (*n* = 672)	99.4 (98.5–99.8)		0.64 (0.62–0.67)		69.9 (66.3–73.4)	

*OR* Odds Ratio (reference: Baseline), *CI* Confidence Interval

***continuity correction (Haldane-Anscombe) applied

### Sources of ITNs

At baseline, prior to the distribution, the 713 surveyed households owned a total of 2011 ITNs. According to owner reports, 70.3% (*n* = 1414) originated from previous mass distributions, and 10.0% (*n* = 201) from continuous NMCP distributions that primarily target women attending antenatal care services. An additional 7.5% (*n* = 151) had been purchased in shops, 3.0% (*n* = 61) were received from relatives, 1.9% (*n* = 39) came from NGOs or churches, and the remaining 5.9% (*n* = 119) were obtained from other or unspecified sources.

At follow-up, five to seven months after the mass distribution, the 672 surveyed households owned 2283 ITNs. Of these, 87.7% (*n* = 2002) were reported to originate from any previous mass distribution, and 3.3% (*n* = 75) from continuous NMCP distribution. A further 1.5% (*n* = 35) had been purchased in shops, 1.5% (*n* = 34) came from relatives, 1.1% (*n* = 26) were obtained from NGOs or churches, and for 4.6% (*n* = 106) the source could not be recalled by owners.

### Distribution targets

The ITN distribution substantially altered the composition of nets present in households, with many older nets replaced by newly distributed ITNs and a large proportion of older nets lost to follow-up. The proportion of ITNs matched to the most recent mass distribution at both baseline and follow-up, both overall and stratified by province, is summarised in Table 2. Imputation using ITN product (brand), manufacturing year, and owner-reported source revealed that overall, at baseline, 46.0% of ITNs (*n* = 913) originated from the previous mass distribution, which had occurred approximately three years earlier. At follow-up, 61.3% of ITNs (*n* = 1395) could be matched to the most recent mass distribution conducted 5–7 months before the survey. Approximately 55.8% (*n* = 1123) of older ITNs present at baseline were discarded or repurposed between the two survey rounds (Table 2) indicating that more than half of all nets in use were replaced by new ITNs within the first six months.

**Table 2:** Proportion of ITNs originating from the previous mass distribution and ITNs lost between baseline and follow-up

Province	Baseline Proportion % (*n/N*)	Follow-up % (*n/N*)	Old ITNs lost % (*n/N*)
Madang	47.9 (306/639)	62.5 (514/823)	51.6 (330/639)
East Sepik	44.9 (293/653)	48.4 (291/601)	52.5 (343/653)
West Sepik	43.7 (314/719)	68.7 (590/859)	62.6 (450/719)
Overall	45.4 (913/2011)	61.1 (1395/2283)	55.8 (1123/2011)

*N* total surveyed nets; *n* number of nets from previous mass distribution

Applying the NMCP’s distribution logic to the observed household composition at baseline yielded an expected target of 2156 ITNs for the 4073 household members—equivalent to one new ITN for every 1.9 people. At follow-up, 1395 ITNs originating from the new mass distribution were observed among 3703 people, corresponding to one new ITN for every 2.7 people.

Table 3 compares the expected number of new ITNs for the observed household member composition, with the observed number of newly distributed ITNs per person at follow-up, including adjustments for 16–23% attrition by six months post-distribution, as documented in previous durability studies in the study area [8]. When these estimates were stratified by province, it became apparent that distribution targets were likely achieved in Madang and West Sepik where only a small shortfall (< 8%) was observed, but not in East Sepik, where a shortfall of around 40% was observed.

**Table 3:** Target new ITN allocation as per distribution logic, observed new ITN allocation including adjustment for attrition, and estimated shortfall in distribution target

Province	Target # new ITNPP	Observed # new ITNPP	Adj. observed # new ITNPP*	Shortfall
Madang	0.53 (650/1223)	0.42 (514/1212)	0.49–0.52	1.9–7.4%
East Sepik	0.56 (667/1193)	0.28 (291/1040)	0.32–0.34	38.4–41.9%
West Sepik	0.51 (839/1657)	0.41 (590/1451)	0.47–0.50	1.2–6.8%
Overall	0.53 (2156/4073)	0.38 (1395/3703)	0.44–0.46	12.5–17.4%

^*^Adjusted for 16–23% attrition occurring between distribution and post-distribution survey [8]

*ITN* Insecticide-treated net, *ITNPP* Insecticide-treated nets per person

### Person-level determinants of ITN usage

ITN usage by province is presented in Table 4. Overall, ITN usage was 76.7% at baseline (pre-distribution) and increased to 83.4% at 5–7 months post-distribution (follow-up). While the distribution led to substantial increases in usage in Madang and West Sepik, the increase observed in East Sepik was marginal.

**Table 4:** ITN usage stratified by province and overall

Province	Baseline % (*n/N*)	Follow-up % (*n/N*)
Madang	73.3 (933/1223)	81.6 (989/1212)
East Sepik	84.6 (1009/1193)	86.0 (894/1040)
West Sepik	71.5 (1184/1657)	83.2 (1207/1451)
Overall	76.7 (3126/4073)	83.4 (3090/3703)

*N* total number of people surveyed; *n* number of people that slept under a net

*ITN* Insecticide treated net

**Fig. 3:**
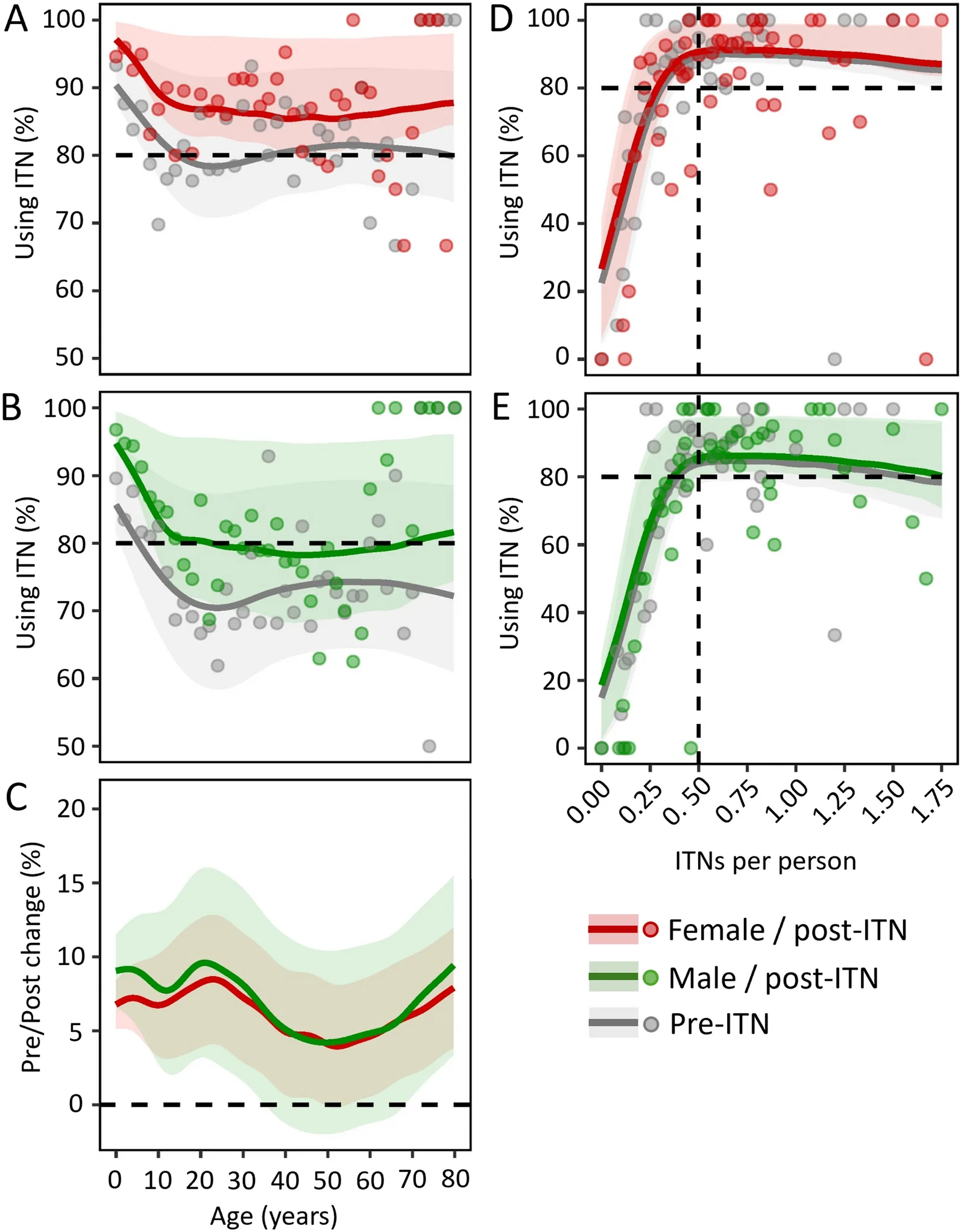
Nonlinear associations between individual insecticide treated net (ITN) use and age or ITNs per person (ITNPP). Red curves and points represent females, green curves and points represent males, and grey represent pre-distribution observations for the respective gender shown in a panel. Circles show raw binned means (2-year bins for age; 0.01-unit bins for ITNPP). Shaded bands are interquartile ranges of the posterior distributions, and solid lines are median posterior predictions from the Bayesian mixed-effects model. **A**, **B**: relationship between age and ITN use for females (**A**) and males (**B**). **C**: estimated percent change in ITN use after distribution relative to before, shown separately for females (red) and males (green) across age. **D**, **E**: relationship between ITNPP and ITN use for females (**D**) and males (**E**). The vertical dashed line marks ITNPP = 0.5 (universal coverage threshold), and the horizontal dashed line marks 80% ITN use

The person-level multivariate model revealed that the ITN distribution substantially increased the odds of ITN use (*OR* = 2.39, 95% *CI:* 1.19–4.81, P* = 0.015, Table 5) across all age groups and for males and females alike (Fig. 3 A–B). Children, younger adults (< 30 years) and older people (70 +) tended to experience the strongest increases in ITN use (Fig. 3 A–B). There was no evidence that the ITN distribution modified gender-specific usage patterns (Fig. 3C).

**Table 5:** Multivariate model estimates of ITN use (fixed effects)

Predictor	*OR* (95% *CI*)/effect*	PTAP*
Post vs pre-distribution (ref.: pre-distribution)	2.39 (1.19–4.81)	0.015
Male vs female (ref. female)	0.50 (0.42–0.59)	< 0.001
Household size (per additional person)	0.93 (0.90–0.98)	0.004
Age (years; spline, Fig. 3A–C)	nonlinear decrease	< 0.001 (global)
ITNs per person (spline, Fig. 3D–E)	nonlinear increase	< 0.001 (global)

^*^Odd ratios (*OR*) and 95% credible intervals (*CI*) are shown for linear fixed effects. Bayesian posterior tail-area probabilities (PTAP) for spline predictors represent a joint (global) test of all spline coefficients. Full model output provided in Table S3 and S4

*ITN* Insecticide treated net, *ref.* Reference category

ITN use varied by gender, age and household size. Males were significantly less likely to use ITNs than females (*OR* = 0.50, 95% *CI:* 0.42–0.59, P* < 0.001, Table 5), and ITN use declined with increasing household size (*OR* = 0.93 per additional person, 95% *CI*: 0.90–0.98, P* = 0.004, Table 5). Age showed a strong nonlinear association with ITN use, with children (0–12 years) much more likely to use ITNs than adults (global P* < 0.001, Table 5; Fig. 3 A–B).

The strongest determinant of ITN use was the number of ITNs per person per household (ITNPP). Usage increased steeply at low ITNPP, but the effect plateaued once ITNPP approached ~ 0.5—consistent with the operational definition of universal coverage (one ITN per two people, Fig. 3 D–E). There was no detectable interaction between ITNPP and survey time-point, indicating that the relationship between ITN availability and use did not differ before versus after the campaign.

Substantial heterogeneity in ITN use existed across households, villages, and individuals. Household-level variability was largest [standard deviation (SD) = 1.52, 95% *CI:* 1.35–1.70], followed by village-level variability (SD = 0.68, 95% *CI:* 0.37–1.19) and individual variability (SD = 0.13, 95% *CI:* 0.01–0.36).

**Fig. 4:**
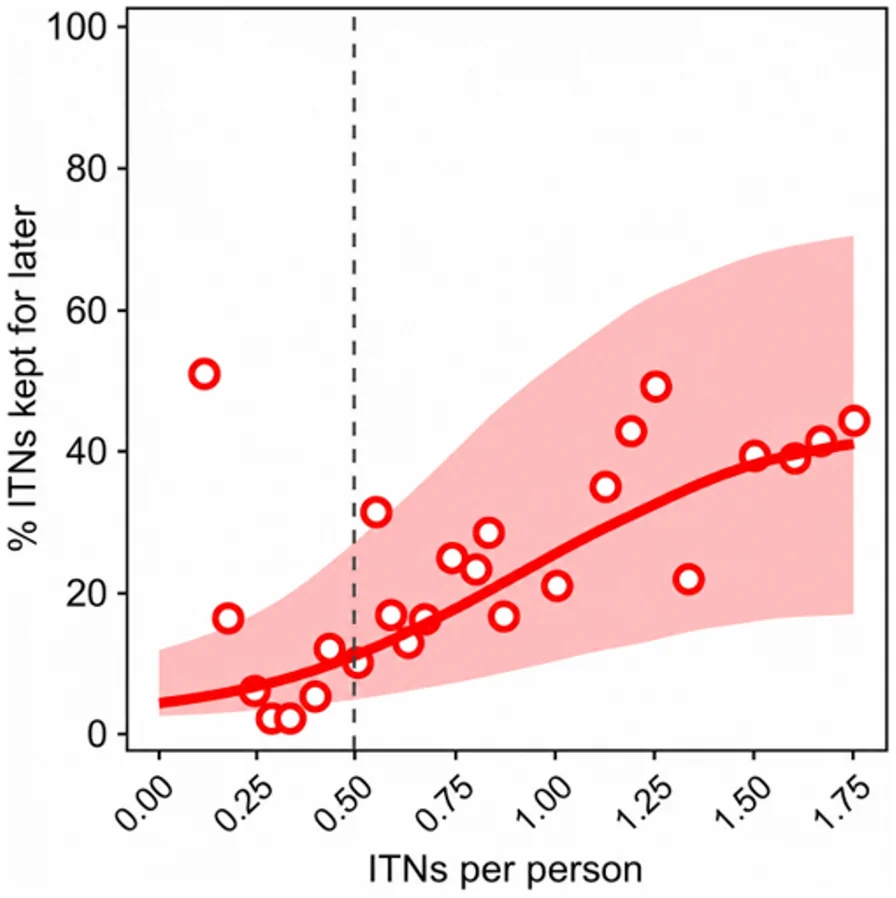
Percentage of insecticde treate nets (ITNs) in a household reserved for later use. Circles (red) are raw data averages, rounded and binned to 0.01 unit intervals. The line and shaded areas is the quasibinomial generalised additive model with 95% confidence band, fit to the raw data. Model specifications are provided in the Supplementary file 1

Figure 4 shows nonlinear associations of ITN kept for later use with ITNPP. The number of new ITNs reserved for later use or reserved for visitors (owner-indicated at follow-up only) increased gradually and significantly (*P* < 0.001) with the number of ITNs available in the household, such that at ITNPP = 1 nearly 25% of all ITNs were reserved for later.

### Person-level determinants of ITN sharing

Overall, 78.6% of people using ITNs shared a net at both baseline (*n* = 2456/3126) and follow-up (*n* = 3090/3928). The proportions of ITN users who shared a net were also very similar across provinces, ranging from 76 to 82% at both time points. The average number of people sharing a single used ITN was 1.90 (95% *CI:* 1.85–1.94) at baseline and 1.89 (95% *CI:* 1.84–1.94) at follow-up, and fewer than 5% of ITN users shared a net with three or more other individuals.

**Fig. 5:**
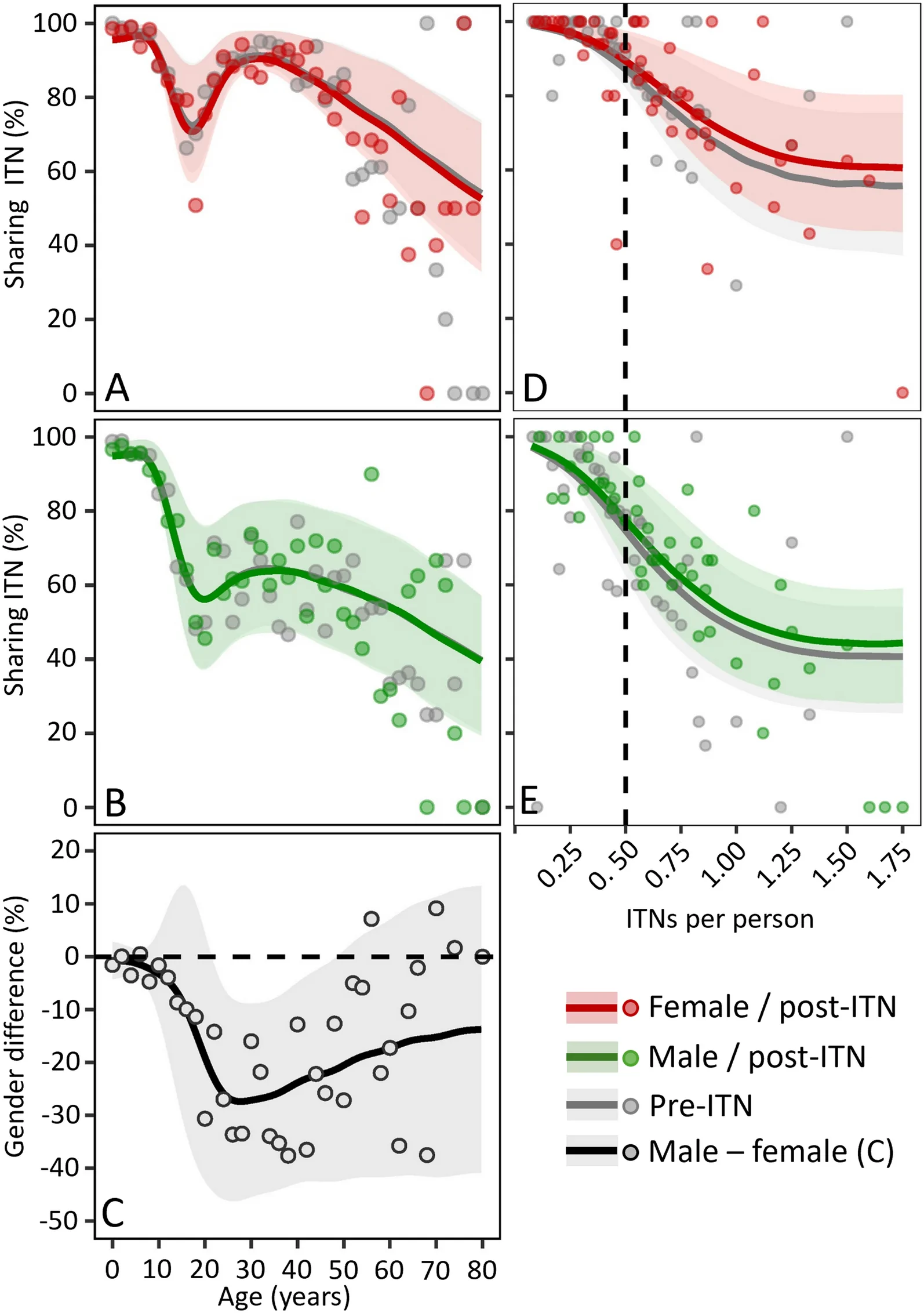
Nonlinear associations between insecticide treated net (ITN) sharing, age, and the number of ITNs per person (ITNPP). Red curves and points represent females; green represent males; grey points show pre-distribution observations for the gender displayed in each panel. Circles show raw binned means (2-year bins for age; 0.01-unit bins for ITNPP). Solid lines show median posterior predictions from the Bayesian mixed-effects model, and shaded bands represent interquartile credible intervals. **A**, **B**: Age-related patterns in ITN sharing for females (**A**) and males (**B**). **C**: Age-related estimated male–female difference (percentage-point difference) in ITN sharing, averaged across pre- and post-distribution data. **D**, **E**: Associations between ITNPP and ITN sharing for females (**D**) and males (**E**). The vertical dashed line marks ITNPP = 0.5 (universal-coverage threshold), and the horizontal dashed line marks 80% sharing

The association between age and ITN sharing was nonlinear and strongly gender specific. The overall main effect of gender was non-significant because differences between males and females were expressed primarily through the age × gender interaction (Table 6). Boys and girls under approximately 12 years shared ITNs at consistently high rates (Fig. 5A, B). Sharing then declined sharply through adolescence and early adulthood (Fig. 5A, B). Among males, sharing generally remained low and gradually decreased with age (Fig. 5B, whereas among females sharing increased again throughout the childbearing years before declining at older ages (Fig. 5B). Consequently, adult men were approximately 20–30 percentage points less likely to share an ITN than adult women (Fig. 5C).

**Table 6:** Multivariate model estimates of ITN sharing

Predictor	*OR* (95% *CI*)/effect*	PTAP**
Post vs pre-distribution (ref.: pre-distribution)	1.31 (0.94–1.79)	n.s
Male vs female (ref.: female)	1.00 (0.41–2.44)	n.s
Household size (per additional person)	0.99 (0.95–1.03)	n.s
ITNs per person (spline)	strong nonlinear decrease	< 0.001 (global)*
Age (years; spline)	complex nonlinear association	< 0.001 (global)*
Age × Gender	nonlinear interaction	0.01–0.03 (global)*

^*^Odd ratios (*OR*) and 95% credible intervals (*CI*) are shown for linear fixed effects

^**^Posterior tail-area probabilities (PTAP) for spline predictors represent a joint (global) test of all spline coefficients. Full model output provided in Tables S5 and S6

*n.s.* not significant, *ITN* Insecticide treated net

Household size did not influence ITN-sharing behaviour (*OR* 0.99, P* = n.s.; Table 6). ITN-sharing behaviour was not directly affected by the mass distribution (*OR* 1.31, P* = n.s.), but was strongly associated with ITN availability (ITNPP, global P* < 0.001). When ITNPP was below the universal coverage threshold (0.5 ITNs per person), more than 80% of ITN users shared a net, whereas sharing declined steadily at higher ITNPP values (Fig. 5D, E).

## Discussion

The results of this study indicate that ITN coverage and usage remain high in some of the highest malaria burden provinces of PNG more than 15 years after the start of the first mass ITN distributions [13]. Across the three study provinces, over 90% of households still owned at least one ITN shortly before the end of the three-year distribution interval, 60% achieved universal coverage, and person-level usage averaged 77%. These values are higher than most previously reported for this region [12, 13, 16–18], and other parts of PNG [14, 19], and reflect the continued operational success of the PNG NMCP’s ITN distribution program.

Generally, the mass distribution further increased all household-level ITN indicators, including the proportion of households with at least one ITN (94.8–99.4%, *P* < 0.001), universal coverage (60.0–69.9%, *P* = 0.002), and ITNs per person (0.55–0.64, *P* < 0.001). After distribution, person-level usage rose from 76.7% to 83.4% (*P* = 0.015). When the results were stratified by province, the same improvement trends were observed in Madang and West Sepik. In contrast, these improvements were not observed in East Sepik Province, where households owned substantially fewer new ITNs than expected 5–7 months after distribution. Correspondingly, household-level indicators and person-level usage in this province showed little change (84.6% vs. 86.0%).

In Madang and West Sepik provinces, the number of ITNs observed in households aligned closely with expectations based on the program’s distribution logic, after considering net attrition [8]. This suggests the NMCP distribution targets were effectively achieved. The high deficit (41.3–44.6%) in new ITNs observed in East Sepik Province, however, highlights the importance of post-distribution assessments. Although poor road access can impede delivery, the study villages in East Sepik were not less accessible than those of the other two provinces, implying that other unidentified factors contributed to the shortfall. These factors may include inaccurate pre-distribution quantification of ITNs (e.g., due to inaccurate census data); misappropriation of ITNs during distribution, or a very rapid loss of ITNs from households post distribution. The present study was not designed to elucidate these factors.

The distribution also resulted in substantial household ITN turnover. Approximately 56% of older ITNs in use before the campaign were discarded or repurposed and replaced with new nets. At the same time, a sizeable proportion of new nets was still being stored for later use, while one third of previously owned ITNs remained in use. These behaviours imply that users consider roughly two thirds of ITNs to have reached the end of their usable lifespan while retaining one third as still functional—consistent with physical durability assessments showing that about half of ITNs become too torn after three years in this region [8].

A key finding is that ITN usage becomes largely independent of per-person coverage once the number of ITNs per person (ITNPP) exceeds the universal coverage threshold (0.5 ITNs per person) [20, 21]. Beyond this point, distributing additional nets is unlikely to increase usage further. However, maintaining universal coverage is essential, as usage drops sharply once ITNPP falls below 0.5. Therefore, in this study area, the three-year distribution interval appears adequate for sustaining universal coverage and near-maximum ITN usage, and shortening the distribution interval to two years does not seem warranted if distribution quality and coverage remain high [22]. Ensuring that distributed ITNs maintain insecticidal efficacy for at least three years is also critical, as several currently available products fall short of this requirement [4, 8, 23, 24]. Distributing ITNs beyond universal coverage may still be advantageous for increasing access—particularly among young adults, a key malaria risk group who are least likely to share ITNs—and for providing a buffer in case future distributions are delayed by operational or other challenges.

Age and gender differences in ITN usage observed here are consistent with previous findings from PNG [17]. Children under 12 years of age are typically the best protected population group. Usage declines through adolescence for males and females, with adolescent males and females increasingly unprotected and at greater risk of mosquito bites [25]. Women of childbearing age exhibit higher usage, reflecting both cultural norms (sharing nets with young children) [26, 27] and continued receipt of ITNs during antenatal care [28]. Men, by contrast, more often claim nets for individual use or forgo them entirely [29]. The mass distribution increased usage similarly across age groups and for both genders, but did not fundamentally alter usage or sharing behaviours, which are strongly shaped by socio-cultural norms. This observation suggests that improving ITN use among demographic groups with lower utilisation would benefit from targeted behavioural change communication interventions, which are currently not widely implemented in PNG.

ITN indicators derived in this study differ substantially from those reported in the 2022–2023 MIS survey, which was conducted 6–18 months earlier in the same provinces [15]. The MIS reported markedly lower household ITN ownership, universal coverage, and usage than observed here. For example, MIS usage estimates ranged from 11.6% to 49.7%, whereas this study measured 71.5–84.6% immediately prior to the subsequent distribution—when ITN availability is expected to be lowest within the distribution cycle and nets are at their maximum age. Similarly, MIS household ITN ownership estimates (34–68%) contrasted sharply with our findings (90–97% at three years post-distribution), suggesting that differences in survey timing are unlikely to explain the observed discrepancies.

Differences in survey methodology and sampling may partly account for these inconsistencies. The present study sampled a larger number of households per village (60 vs. 30 in the MIS), resulting in more precise village-level estimates but in only four villages per provinces whereas the MIS is conducted in 5 villages per province. The present study thus resulted in a much larger dataset for each province as compared to the MIS. While household selection procedures were near identical, the studies sampled different villages. All study sites were located in low-altitude areas (< 305 m) and were eligible for ITN distribution, and we are not aware of any major geographical or sociocultural differences that could plausibly explain the systematic differences in ITN indicators. Moreover, the relatively limited heterogeneity observed in our dataset suggests that sampling variation alone is unlikely to account for these findings.

A key methodological distinction is that the MIS relies on self-reported ITN ownership and usage, whereas this study included direct inspection of ITNs. This makes overestimation of ITN availability in the present study unlikely, although some underestimation cannot be excluded if households withheld nets from inspection. In contrast, self-reported data in the MIS may be subject to both under- and over-reporting. One possible explanation for the consistently lower MIS estimates is systematic under-reporting of ITNs by respondents, potentially due to expectations of receiving new nets. Regardless of the underlying causes, these discrepancies highlight the need for cautious interpretation of ITN indicators derived from different survey methodologies, particularly given their importance in informing national malaria control strategies.

The present study had important limitations. No a priori sample size calculation was conducted, as this study was designed as an operational evaluation embedded within routine ITN distribution activities rather than a hypothesis-driven trial. It was limited to three very high burden provinces where ITN use is traditionally high. The results can thus not be generalised to areas of PNG where ITN use is traditionally low such as many of the island provinces. Further studies are needed to understand the impact of ITN distributions on ITN indicators in these areas. The aforementioned specific methodological differences may limit the comparability to previous surveys to some extent, however the overall very similar study design and methods still warrant this comparison, while acknowledging the limitations. The cross-sectional and partially overlapping nature of the baseline and follow-up surveys required empirical record linkage but may have reduced the risk of behavioural bias associated with repeated household visits.

## Conclusions

Overall, this study shows that in PNG provinces accounting for nearly half of the national malaria burden, ITN coverage and usage remain higher than recently suggested. The primary effect of distribution campaigns in these settings is to replace worn nets with newer ones, thereby sustaining protection and maintaining access to nets that may offer greater insecticidal efficacy. A triennial distribution cycle appears sufficient to maintain high coverage and usage rates. Further increases in use are likely to require targeted behavioural change communication interventions. Post-distribution surveillance remains critical to ensure that distribution targets are achieved. However, it is essential that distributed ITNs retain full efficacy against pyrethroid-susceptible vectors, as reduced insecticidal durability could undermine the effectiveness of an otherwise well-functioning national ITN distribution programme.

## Supplementary Information

Supplementary Material 1.Table S1: Raw data for number of households, number of people and number of Insecticide treated netsper province at baseline and follow-up. Table S2: Exact survey dates for the pre- and post-distribution surveys per village. Table S3: ITN use model multilevel hyperparameters. Table S4. ITN use model regression coefficients. Table S5. ITN sharing model multilevel hyperparameters. Table S6. ITN sharing model regression coefficients. Table S7. ITNs ‘kept for later’ model regression coefficients

## Data Availability

Data that supports the findings of this study are available from the corresponding author upon reasonable request.

## Abbreviations

*CI*: Confidence or credible interval
DOF: Degrees of freedom
ITNPP: ITNs per person
ITNs: Insecticide-treated nets
MIS: Malaria Indicator Survey
NGO: Non-governmental organisation
NMCP: National Malaria Control Program
OR: Odds ratio
PNG: Papua New Guinea
PNGIMR: Papua New Guinea Institute of Medical Research
PTAP: Posterior tail area probability
RAM: Rotarians Against Malaria
WHO: World Health Organization
