# Supplementary material for: Impact of mass distribution on insecticide-treated net coverage and usage in three high burden malaria provinces of Papua New Guinea
Source: Infect Dis Poverty. 2026 Sep 14;15:103. doi: 10.1186/s40249-026-01494-x (PMC13573335; doi:10.1186/s40249-026-01494-x)
Supplement: Supplementary file 1 [file 40249_2026_1494_MOESM1_ESM.docx]

## Supporting Information

## Table S1: Raw data for number of households, number of people and number of Insecticide treated nets (ITNs) per province at baseline and follow–up. This table corresponds to Table 1 in the main text and enables the calculation of the presented univariate odds ratios (*OR*) and ITNs per person (ITNpp) values.

| **Province** | **Timepoint** | ***N* Households** | ***N***  **people** | ***N***  **ITNs** | ***N* (%)**  **At least 1 ITN** | ***N* (%)**  **Universal Coverage** |
| --- | --- | --- | --- | --- | --- | --- |
| East Sepik | Baseline | 239 | 1193 | 653 | 232 (97.1%) | 168 (70.3%) |
|  | Follow–up | 215 | 1040 | 601 | 212 (98.6%) | 143 (66.5%) |
| Madang | Baseline | 234 | 1223 | 639 | 228 (97.4%) | 146 (62.4%) |
|  | Follow–up | 234 | 1212 | 823 | 233 (99.6%) | 177 (75.6%) |
| West Sepik | Baseline | 240 | 1657 | 719 | 216 (90.0%) | 114 (47.5%) |
|  | Follow–up | 223 | 1451 | 859 | 223 (100.0%) | 150 (67.3%) |
| Overall | Baseline | 713 | 4073 | 2011 | 676 (94.8%) | 428 (60.0%) |
|  | Follow–up | 672 | 3697 | 2283 | 668 (99.4%) | 470 (69.9%) |

ITN Insecticide treated net

Table S2: Exact survey dates for the pre- and post-distribution surveys per village.

| **Province** | **District** | **Village** | **Baseline** | | **Follow–up** | | **Month of ITN Distribution** |
| --- | --- | --- | --- | --- | --- | --- | --- |
|  |  |  | **Start** | **End** | **Start** | **End** |  |
| East Sepik | Wewak | Banak | 29/11/2023 | 1/12/2023 | 21/10/2024 | 24/10/2024 | Mar-24 |
| East Sepik | Maprik | Ilahup | 28/11/2023 | 30/11/2023 | 22/10/2024 | 25/10/2024 | Mar-24 |
| East Sepik | Maprik | Serikum | 23/11/2023 | 25/11/2023 | 14/10/2024 | 17/10/2024 | Mar-24 |
| East Sepik | Maprik | Yilehina | 23/11/2023 | 25/11/2023 | 14/10/2024 | 17/10/2024 | Mar-24 |
| Madang | Bogia | Aidibal | 30/10/2023 | 1/11/2023 | 4/06/2024 | 7/06/2024 | Dec-23 |
| Madang | Rai Coast | Kaliku | 16/10/2023 | 18/10/2023 | 6/05/2024 | 9/05/2024 | Nov-23 |
| Madang | Madang | Kananam | 2/10/2023 | 4/10/2023 | 1/07/2024 | 4/07/2024 | Jan-24 |
| Madang | Madang | Soh | 21/09/2023 | 23/09/2023 | 9/07/2024 | 12/07/2024 | Jan-24 |
| West Sepik | Vanimo Green | Ossima | 24/01/2024 | 26/01/2024 | 19/08/2024 | 21/08/2024 | Feb-24 |
| West Sepik | Vanimo Green | Samunai | 31/01/2024 | 2/02/2024 | 27/08/2024 | 30/08/2024 | Feb-24 |
| West Sepik | Aitape Lumi | Ulau | 2/02/2024 | 5/02/2024 | 29/08/2024 | 1/09/2024 | Mar-24 |
| West Sepik | Vanimo Green | Yako | 24/01/2024 | 26/01/2024 | 19/08/2024 | 22/08/2024 | Feb-24 |

ITN Insecticide treated net

## ITN use model formulation

The statistical model used for ITN use is given in equation S1.

$logit(P(Y_{ijkm} = 1)) = f₁({ITNPP}_{ijkm}) + f₂({Age}_{ijkm}) + \beta_{TP}\cdot{TP}_{ijkm} + f_{3}({Age}_{ijkm})\times{TP}_{ijkm} +\beta_{HHSize}\cdot{HHSize}_{ijkm} + \beta_{Gender}\cdot{Gender}_{ijkm} + {b_{0k}}^{(V)} + {b_{1k}}^{(V)}\cdot{TP}_{ijkm} + {b_{0j}}^{(H)} + {b_{0m}}^{(P)}$

**Equation S1: Statistical model developed for ITN use**

With,

- Outcome (*Y*): 1 = slept under an ITN, 0 = did not
- ITNs per person (*ITNPP*): natural cubic spline with 3 degrees of freedom *f_1_*
- *Age*: natural spline with 4 degrees of freedom *f_2_*
- Survey Time Point (*TP*): Baseline and Follow-up
- Household size (*HHSize*) Numeric, number of people in household
- *Gender*: Levels: Male and female
- Interaction between *Age* and *TP,* *f_3_*
- Random intercepts for villages and random slopes for *TP*: ${b_{0k}}^{(V)} + {b_{1k}}^{(V)}$
- Random intercepts for households: ${b_{0j}}^{(H)}$
- Random intercepts for people: ${b_{0m}}^{(P)}$

The regression parameters resulting from the model fit to the data are given in Table S3 and S4.

Table S3: ITN use model multilevel hyperparameters (random effects)

| **Parameter** | **Estimate (95% *CI*)** | **PTAP** |
| --- | --- | --- |
| sd(Household Intercept) | 1.52 (1.35, 1.70) | <0.001 |
| sd(Person Intercept) | 0.13 (0.01, 0.36) | <0.001 |
| sd(Village Intercept) | 0.68 (0.37, 1.19) | <0.001 |
| sd(Village TP slope) | 0.35 (0.06, 0.72) | <0.001 |
| cor(Village Intercept, TP slope) | 0.22 (–0.49, 0.82) | 0.544 |

ITN Insecticide treated net; CI Confidence interval; PTAP posterior tail–area probability

**Table S4: ITN use model regression coefficients (fixed effects)**

| **Coefficient** | **Estimate (95% *CI*)** | **PTAP** |
| --- | --- | --- |
| Intercept | –0.12 (–0.94, 0.73) | 0.761 |
| ITNpp_ns_1_* | 3.09 (2.50, 3.69) | <0.001 |
| ITNpp _ns_2_* | 9.17 (7.95, 10.38) | <0.001 |
| ITNpp _ns_3_* | 2.13 (1.02, 3.28) | <0.001 |
| Age_ns_1_* | –1.74 (–2.24, –1.25) | <0.001 |
| Age _ns_2_* | –0.53 (–1.18, 0.11) | 0.110 |
| Age _ns_3_* | –2.50 (–3.59, –1.39) | <0.001 |
| Age _ns_4_* | –1.02 (–2.30, 0.25) | 0.117 |
| TP (Follow-up) | 0.87 (0.17, 1.57) | 0.013 |
| HHSize | –0.07 (–0.11, –0.02) | 0.002 |
| Gender (male) | –0.69 (–0.86, –0.52) | <0.001 |
| Age_ns_1_* × TP (Follow-up) | –0.32 (–1.07, 0.41) | 0.396 |
| Age_ns_2_* × TP (Follow-up) | –1.17 (–2.07, –0.30) | 0.006 |
| Age_ns_3_* × TP (Follow-up) | –1.34 (–2.86, 0.15) | 0.08 |
| Age_ns_4_* × TP (Follow-up) | 0.39 (–1.20, 2.08) | 0.65 |

*spline parameters; ITN Insecticide treated net; CI Confidence interval; PTAP posterior tail–area probability

## ITN sharing model formulation

The statistical model used for ITN sharing is given in equation S2.

$logit(P(Y_{ijkm} = 1)) = f₁({ITNPP}_{ijkm}) + f₂({Age}_{ijkm}) + \beta_{TP}\cdot{TP}_{ijkm} + f_{3}({Age}_{ijkm})\times{Gender}_{ijkm} +\beta_{HHSize}\cdot{HHSize}_{ijkm} + \beta_{Gender}\cdot{Gender}_{ijkm} + {b_{0k}}^{(V)} + {b_{1k}}^{(V)}\cdot{TP}_{ijkm} + {b_{0j}}^{(H)} + {b_{0m}}^{(P)}$

**Equation S2: Statistical model developed for ITN sharing**

With,

- Outcome (*Y*): 1 = shared ITN with at least one other person, 0 = did not
- ITNs per person (*ITNPP*): natural cubic spline with 2 degrees of freedom *f_1_*
- *Age*: natural spline with 5 degrees of freedom *f_2_*
- Survey Time Point (*TP*): Baseline and Follow-up
- Household size (*HHSize*) Numeric, number of people in household
- *Gender*: Levels: Male and female
- Interaction between *Age* and *Gender,* *f_3_*
- Random intercepts for villages and random slopes for *TP*: ${b_{0k}}^{(V)} + {b_{1k}}^{(V)}$
- Random intercepts for households: ${b_{0j}}^{(H)}$
- Random intercepts for people: ${b_{0m}}^{(P)}$

The regression parameters resulting from the model fit to the data are given in Table S5 and S6.

Table S5: ITN sharing model multilevel hyperparameters (random effects)

| **Parameter** | **Estimate (95% *CI*)** | **PTAP** |
| --- | --- | --- |
| sd(Household Intercept) | 0.76 (0.58, 0.94) | <0.001 |
| sd(Person Intercept) | 1.26 (0.96, 1.56) | <0.001 |
| sd(Village Intercept) | 0.15 (0.01, 0.38) | <0.001 |
| sd(Village TP slope) | 0.34 (0.05, 0.70) | <0.001 |
| cor(Village Intercept, TP slope) | -0.05 (–0.79, 0.78) | 0.901 |

ITN Insecticide treated net; CI Confidence interval; PTAP posterior tail–area probability

Table S6: ITN sharing model regression coefficients (fixed effects)

| **Coefficient** | **Estimate (95% *CI)*** | **PTAP** |
| --- | --- | --- |
| Intercept | 7.42 (6.44, 8.43) | <0.001 |
| ITNpp_ns_1_* | –9.26 (–10.40, –8.09) | <0.001 |
| ITNpp_ns_2_* | –3.68 (–4.60, –2.77) | <0.001 |
| Age_ns_1_* | –4.40 (–5.24, –3.60) | <0.001 |
| Age_ns_2_* | –0.16 (–1.04, 0.71) | 0.698 |
| Age_ns_3_* | –3.18 (–4.11, –2.29) | <0.001 |
| Age_ns_4_* | –2.92 (–4.51, –1.35) | <0.001 |
| Age_ns_5_* | –5.56 (–7.02, –4.12) | <0.001 |
| Gender (male) | –0.03 (–0.85, 0.78) | 0.950 |
| TP (Follow-up) | 0.25 (–0.05, 0.53) | 0.088 |
| HHSize | 0.01 (–0.02, 0.05) | 0.483 |
| Age_ns_1_* × Gender (male) | –0.10 (–1.11, 0.92) | 0.833 |
| Age_ns_2_* × Gender (male) | –3.03 (–4.09, –1.96) | <0.001 |
| Age_ns_3_* × Gender (male) | –0.55 (–1.71, 0.58) | 0.362 |
| Age_ns_4_* × Gender (male) | –1.20 (–3.04, 0.65) | 0.208 |
| Age_ns_5_* × Gender (male) | –0.54 (–2.33, 1.24) | 0.552 |

* spline parameters; ITN Insecticide treated net; CI Confidence interval; PTAP posterior tail–area probability

## Proportion of ITNs ‘kept for later’ model formulation

The statistical model for the proportion of ITNs reserved for later us is given below.

Let:

- *Y_i_* = proportion of ITN retained for later use for observation *i*
- *p_i_* = expected probability of ITN retained for later use
- *ITNPP_i_* = ITNs per person for observation i

The GAM is:

$$Y_{i} \sim quasi-binomial (p_{i},\Phi)$$

With:

$$logit(p_{i}) = \beta_{0} + f({ITNPP}_{i})$$

Where:

- *β_0_* is the intercept,
- *f ()* is a smooth function estimated using a penalised thin-plate regression spline with 5 basis dimensions,
- *ϕ* is the dispersion parameter.

The regression parameters resulting from the model fit to the data are given in Table S7.

Table S7: ITNs ‘kept for later’ model regression coefficients

| **Term** | **Estimate (95% CI) / EDF*** | ***P*** |
| --- | --- | --- |
| Intercept (β₀) | –1.99 (–2.17, –1.80) | < 0.001 |
| s(ITNPP) | EDF = 2.843 | < 0.001 |

*EDF: Effective degrees of freedom; ITN Insecticide treated net; CI Confidence interval;

**Survey Instrument**

The survey instrument was implemented in Epicollect 5 (https://five.epicollect.net/). The study used a hierarchical survey form structure. In each cross sectional survey, each household is represented by one <Basic Household Information Form> and as many <Household Member Information> and <ITN Information> forms as there are household members and ITNs in a specific household. Other information was also collected in the same survey which is not presented here as it is not subject of this manuscript.

Figure 1: Survey Instrument Structure. Hierarchical forms were used for each household enabling entry of a variable number of instances of household member and ITN information.

**<Basic Household Information Form>**

**Type: Parent Form (not repeatable)**

**Study Code:** Drop Down Village Identifier

**Follow-up Round:** Drop Down Round Identifier

**Household ID:**

**Tablet ID:** Drop Down Table Identifier

**Date of Survey:** Date field

**Province:** Drop Down Province Identifier

**Village:** Drop Down Village Identifier

**Hamlet (if applicable):** free text field

**Name of Household Head:** free text field

**How many structures where people sleep in total?** Integer field

**How many rooms where people sleep (in total)?** Integer field

**Total Number of people living in the household (permanently stay/sleep there):** Integer field

**How many children under 5?** Integer field

**How many people slept in this household last night?** Integer field

**Does this Household have any mosquito nets?** Radio button Yes/No field

**If yes, how many mosquito nets (ITNs) does this household have?** Integer field

**How many ITNs are in their original packaging?** Integer field

**Does this household have (‘household wealth proxies** ): Drop down ‘wealth proxy’ field

**Household GPS Location:** GPS acquisition button

**<Household Member Information Form>**

**Type: Child Form to <Household Information> (infinitely repeatable)**

**Household Member Number:** Integer field

**Household Member Name:** Free text field

**Relation to Household Head: Drop Down ‘Relation to Household Head’ menu**

**Age:** Integer field

**Gender:** Radio button male/female field

**Slept under ITN last night?** Radio button Yes/No field

**If yes, which ITN Number (refer to the ITN number assigned):** Integer field

**Share ITN with how many others?:** Integer field

**At what time. does this household member usually go to bed?:** Time selection field

**<ITN Information Form>**

**Type: Child Form to <Household Information> (infinitely repeatable)**

**ITN Number (assigned by survey team):** Integer field

**Is this a treated net?:** Radio button Yes/No field

**ITN brand:** Drop Down ‘ITN product’ list field

**Manufacture Year of Net:** Integer field

**If other brand, please name:** Free text field

**Do you know when you got this net?:** Radio button Yes/No field

**If yes, when did you get this net (month/year)?:** Date selection field

**How did you get this net?:** Drop Down ‘ITN source list’ field

**If other, please specify:** Free text field

**Did anyone sleep under this net last night?** Radio button Yes/No field

**If yes, who slept under this net (refer to HH member number):** Integer field

**If no, why was this ITN not used?:** Drop Down ‘Reasons for not using’ field

**If other, please describe?:** Free text field

**Has this net ever been washed?:** Radio button Yes/No field

**If yes, how many times has it been washed?** Drop Down ‘Wash Number’ field

**Are there bedbugs on the net?:** Radio button Yes/No field

**Bed bug collected for assessment** Radio button Yes/No field

**If Bedbug was collected, enter Bedbug sample code:** Free text field

**Was this ITN collected and replaced?:** Radio button Yes/No field

**If ITN was collected enter Net sample code:** Free text field

**If ITN was collected enter NNI Household ID and Net ID:** Free text field

**Is the collected ITN tagged with the NNI used Net ID?:** Radio button Yes/No/NA field

**Photograph of ITN label (if possible):** Photo file attached
